# Primary and Secondary Sutures of War Wounds

**Published:** 1918-03

**Authors:** 


**Primary and Secondary Sutures of War Wounds.** By M. GABRIEL POTHERAT, reported by M. Pierre Delbet. Trans. and Abs. from the *Bulletins et Mémoires de la Société de Chirurgie de Paris*, Dec. 18, 1917.

Out of a total of 1005 cases, P. practised primary suture in 221, and secondary suture in 459; in all, 880 sutures. Among the primary sutures there were 2 complete failures due to insufficient excision, and 11 partial failures caused by incomplete hemostasis.

P. states that he never attempts primary suture if there are large seton wounds with an irregular track, or when there is any considerable breakage of bone, or when the injury is more than twelve hours old.

He describes his technique for secondary suture as follows :

" After surgical treatment for the purpose of cleansing, I irrigate the wound continuously or intermittently, or else I apply a moist dressing with a solution of chloride of magnesium (12.5 : 1000) with 0.125 gr. of chloride of ammonium added ".

He prefers this solution to any of the antiseptics used either before or during the war, all of which he has found disappointing. He has found the chloride of magnesium solution easy to prepare, harmless, and especially effective in stimulating the cells By this method he claims to have stopped all suppuration within 8 or 10 days, and to have brought about healthy granulations Within 2 or 3 days the temperature falls to normal. " At this stage, " says the author, " I give the wound a thorough washing and even rub it with one or two liters of a warm chloride of magnesium solution. I effect a secondary suture either surgically, or, in case the lips of the wound come easily in contact, by means of collodionized or leucoplastic bands. " If heliotherapy is added, the results are obtained even more rapidly.

